# *In vivo* evaluation of integrin αvβ6-targeting peptide in NSCLC and brain metastasis

**DOI:** 10.3389/fonc.2023.1070967

**Published:** 2023-03-10

**Authors:** Di Fan, Chengkai Zhang, Qi Luo, Baowang Li, Lin Ai, Deling Li, Wang Jia

**Affiliations:** ^1^ Department of Nuclear Medicine, Beijing Tiantan Hospital, Capital Medical University, Beijing, China; ^2^ Department of Neurosurgery, Beijing Tiantan Hospital, Capital Medical University, Beijing, China; ^3^ Guangzhou Laboratory, Guangzhou International Bio Island, Guangzhou, Guangdong, China

**Keywords:** integrin αvβ6, NSCLC, brain metastases, PET, near-infrared fluorescence imaging

## Abstract

**Introduction:**

Integrin αvβ6, which is upregulated in malignancies and remains absent or weak in normal tissue, is a promising target in molecular imaging therapeutics. In vivo imaging of integrin αvβ6 could therefore be valuable for early tumor detection and intraoperative guidance.

**Methods:**

In this study, integrin αvβ6-targeting probe G2-SFLAP3 was labeled with near-infrared (NIR) dye Cy5.5 or radioisotope 68Ga. The resulting probes were evaluated in integrin αvβ6-positive A549 and αvβ6-negative H1703 xenograft mice models.

**Results:**

The cellar uptake of G2-SFLAP3-Cy5.5 was consistent with the expression of integrin αvβ6. Both subcutaneous and brain metastatic A549 tumors could be clearly visualized by NIR fluorescent imaging of G2-SFLAP3-Cy5.5. A549 tumors demonstrated the highest G2-SFLAP3-Cy5.5 accumulation at 4h post-injection (p.i.) and remain detectable at 84h p.i. The fluorescent signal of G2-SFLAP3-Cy5.5 was significantly reduced in H1703 and A549-blocking groups. Consistently, small-animal PET imaging showed tumor-specific accumulation of 68Ga-DOTA-G2-SFLAP3.

**Discussion:**

G2-SFLAP3 represents a promising agent for noninvasive imaging of non-small cell lung cancer (NSCLC) and brain metastases.

## 1 Introduction

Non-small cell lung cancer (NSCLC) remains the leading cause of cancer death (1). There is an increasing prevalence of brain metastases from NSCLC, and approximately 45% of patients with NSCLC will develop brain metastases during the course of illness (2, 3). Despite recent advances in the treatments, the prognosis of brain metastatic NSCLC was still dismal (4, 5). Moreover, the blood-brain barrier and unique environment in the brain increased the difficulty of diagnosis and treatment (6). Therefore, early detection of NSCLC and their metastases is crucial for treatment planning and prognosis.

Integrin αvβ6 was an epithelia-specific transmembrane glycoproteins (7). It is mostly undetectable in normal tissue; and highly expressed in embryogenesis, wound healing, inflammation, and various epithelial malignancies7. Moreover, the overexpression of integrin αvβ6 is associated with multiple malignant behaviors of cancer, including transforming growth factor-β (TGF-β) activation, epithelial-mesenchymal transition, tumor invasion, and metastasis (7–9). Current studies also showed integrin αvβ6 was upregulated in primary (10, 11), and brain metastatic (12) NSCLC. Given the unique properties of integrin αvβ6, there was growing interest in identifying malignancies by integrin αvβ6 (13).

Several integrin αvβ6-targeting probes have been recently developed and tested for tumor imaging (13). The peptide A20FMDV2, derived from the foot and mouth disease virus, was the first agent developed for integrin αvβ6 binding (14). Then, sunflower trypsin inhibitor (SFTI)-based peptide (SFITGv6) was developed for integrin αvβ6 imaging and showed favorable efficacies in NSCLC (10, 15). After that, SFTI-based peptide containing latency-associated peptide (SFLAP3) (GRGDLGRL) was synthesized and showed better binding potential with integrin αvβ6 than SFITGv6 (16). Nevertheless, studies about newly developed SFLAP3 were limited to imaging for head and neck squamous cell carcinoma (HNSCC) and pancreatic cancer (16, 17). The application of SFLAP3 needed to be extended.

Molecular imaging can assist diagnosis and treatment of malignancies in multimodalities. Near-infrared (NIR) dyes, with excitation and emission wavelength of 700-900nm in NIR-I and 1000nm-1700nm in NIR-II, can be applied in optical fluorescence and guide surgical resection (18, 19). Positron emission tomography (PET) is a highly sensitive, quantitative, and noninvasive technique that has been used for tumor detection. In the current study, we aimed to develop 68Ga-labeled or Cy5.5-labeled peptide G2-SFLAP3 (GGGRGDLGRL), and investigated the imaging effect of G2-SFLAP3 for both subcutaneous and brain-metastatic NSCLC in xenograft mice models.

## 2 Materials and methods

### 2.1 Probe design and synthesis

G2-SFLAP3-Cy5.5 and DOTA-G2-SFLAP3 (**Figure S1**) were prepared by direct conjugation of the G2-SFLAP3 peptide with Cy5.5-NHS and DOTA-NHS. The peptide G2-SFLAP3 was synthesized by Tanzhenbio (Nanchang, China). The 68GaCl3 solution was obtained from a commercially available 68Ge/68Ga generator (ITG Isotope Technologies Garching GmbH, Garching, Germany).

The reversed-phase high-performance liquid chromatography (HPLC) system was the same as that previously described (20). HPLC method (for conjugation): The flow rate was 4.0 mL/min. The mobile phase was isocratic with solvent A (DD Water +0.05% TFA) and solvent B (Acetonitrile +0.05% TFA). The HPLC purity of Cy5.5-NHS and DOTA-G2-SFLAP3 was >95%. And we used mass spectrometry to confirm the identity of the product. The 68Ga-DOTA-G2-SFLAP3 labeling was completed within 30 min, and C18-cartridge purification removed all free 68Ga.

### 2.2 Cell lines and culture condition:

Two human NSCLC cell lines were purchased from the National biomedical experimental cell resource bank, including αvβ6-positive luciferase-expressing A549 (21) and αvβ6-negative H1703 (10).

The A549 cell line was cultured in Dulbecco’s modified Eagle’s medium (Gibico) supplemented with 10% fetal bovine serum and 1% penicillin-streptomycin. The H1703 cell line was cultured in RMPI 1640 (Gibico) supplemented with 10% fetal bovine serum and 1% penicillin-streptomycin. The cells were incubated at 37 ° and 5% CO2.

### 2.3 Immunofluorescence and cell-binding assay

4×10^4^ Cells were inoculated on coverslips into 24-well plates. After being cultured overnight, cells were fixed with paraformaldehyde at room temperature and rinsed with PBS. The cells were incubated with rabbit anti-integrin αvβ6 antibody (1:100, Bioss, bs-1356R) at 37°. The coverslips were then washed three times with PBS and incubated with the secondary antibody (FITC conjugated goat anti-rabbit lgG antibody, Abcam) for 1h at room temperature. After being washed three times with PBS, DAPI staining solution was used for nuclei staining. The result was observed through laser confocal microscope.

For the cell-binding assay, cells were seeded and cultured under the same condition as mentioned above. Then, cells were incubated with 1 umol/L G2-SFLAP3-Cy5.5 for 1h at 37°. In the competitive combination experiment, 100 umo/L G2-SFLAP3 was incubated for 1 hour before adding G2-SFLAP3-Cy5.5. After washing and fixation, coverslips were stained with DAPI and visualized by the laser confocal microscope. All microscopic images were analyzed by Image J (v 1.53).

### 2.4 Tumor model construction

All animal experiments were performed following instructions and permissions of the ethical committee of Beijing Tiantan Hospital. Female Balb/c nude mice (5-week-old) were purchased from Charles River Laboratories, Beijing, China. The subcutaneous tumor models were established by injecting 5×10^6^ cancer cells dissolved in 100μL of PBS subcutaneously into the right shoulder. After 3 to 4 weeks, while tumors grew into 6-8mm, animal models were used for *in vivo* imaging. Brain metastatic tumor models were constructed by stereotactic injection of A549 (5×10^5^ cells dissolved in 5 μL) into the right hemisphere using the following coordinates: 1mm posterior to the bregma, 2mm lateral to the midline, and 3mm depth into the brain surface. Tumor growth was monitored by bioluminescence imaging (IVIS Spectrum, PerkinElmer), after intraperitoneal injection of D-luciferin Potassium (PerkinElmer, 15mg/mL, 0.15mg/g), and the total photon flux (photons/sec) was quantified by Living Image (v4.5.5, PerkinElmer).

### 2.5 NIR fluorescence imaging

To analyze the metabolic characteristic of G2-SFLAP3-Cy5.5, 1noml G2-SFLAP3-Cy5.5 dissolved in 200 μL PBS was injected into A549 tumor-bearing mice through tail vein (n=4); meanwhile, fluorescence images were captured at 1h to 84h post-injection (p.i.) by IVIS Spectrum (PerkinElmer, excitation/emission:675/720, binning=4, f-stop=4, exposure time=1s). To analyze the specific binding of G2-SFLAP3-Cy5.5, we injected A549 and H1703 tumor-bearing mice with an equivalent dose of G2-SFLAP3-Cy5.5 (n=4 each group); and performed imaging 2 hours p.i. For the competitive blocking experiment, 500 μg/mouse G2-SFLAP3 was injected 1 hour before the injection of G2-SFLAP3-Cy5.5. Brain metastasis mice models were euthanized 2h after G2-SFLAP3-Cy5.5 injection; *ex vivo* images were captured immediately.

### 2.6 Micro-PET imaging

Tumor-bearing mice were injected with 100 μCi 68Ga-DOTA-G2-SFLAP3 through the tail vein. At 20 min p.i., mice were anesthetized with isoflurane and performed the micro PET imaging (SuperNova, PINGSENG Healthcare). PET images were acquired for 10 minutes and reconstructed with attenuation correction. In the block experiment, the mouse was co-injected with an extra amount (500 μg) of non-radiolabeled G2-SFLAP3.

### 2.7 Statistical analyses

Quantitative data was reported as mean ± SD. Independent student t-tests were used to evaluate the statistical difference. Statistical analyses were performed using Graphpad Prism (Version 9.2.0). A two-tailed p<0.05 was considered statistically significant.

## 3 Results

### 3.1 G2-SFLAP3-Cy5.5 detects integrin αvβ6 expression in Vitro

**Figure 1:**
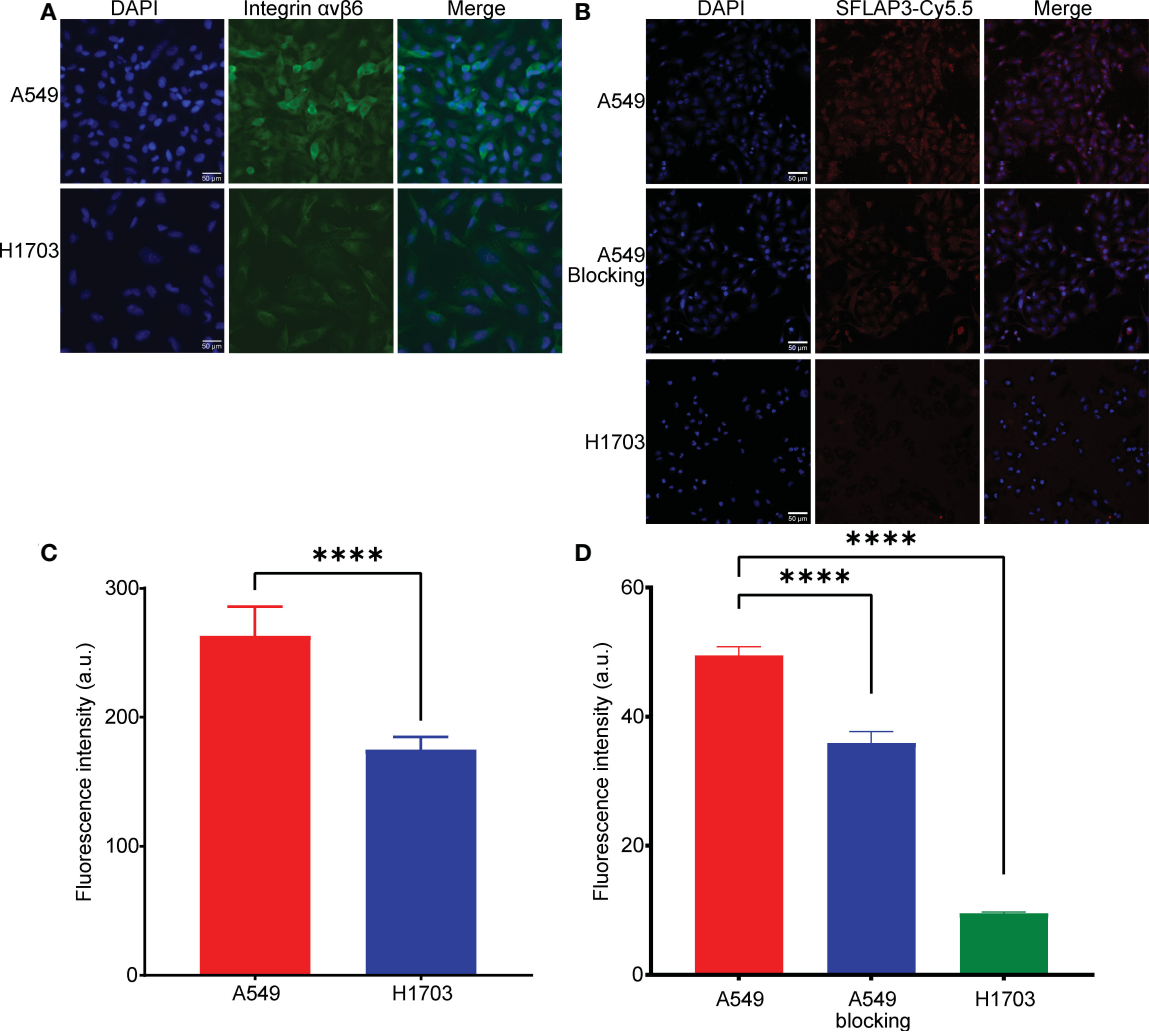
Expression of integrin αvβ6 and uptake of G2-SFLAP3-Cy5.5 in cell lines. **(A)** Immunofluorescence stain of integrin αvβ6 in A549 and H1703 cell lines. **(B)** Cell binding images of G2-SFLAP3-Cy5.5 in A549, H1703, and G2-SFLAP3-blocked A549. **(C, D)** The corresponding average fluorescence intensity of immunofluorescence and cell binding assay, respectively (n=3). Data were presented as mean± SD, ****p < 0.0001.

The expression of integrin αvβ6 was validated by immunofluorescence. As shown in **Figure 1A**, A549 highly expressed integrin αvβ6, while H1703 poorly expressed integrin αvβ6. The expression difference reached a statistical difference (**Figure 1C**). In accordance with integrin αvβ6 expression, A549 showed an intense fluorescent signal after incubation of G2-SFLAP3-Cy5.5, whereas H1703 showed almost no fluorescent signal. The signal intensity was significantly reduced in A549 cells after being blocked by excessive non-fluorescent G2-SFLAP3 (**Figures 1B, D**).

### 3.2 *In vivo* and *ex vivo* fluorescence imaging

**Figure 2:**
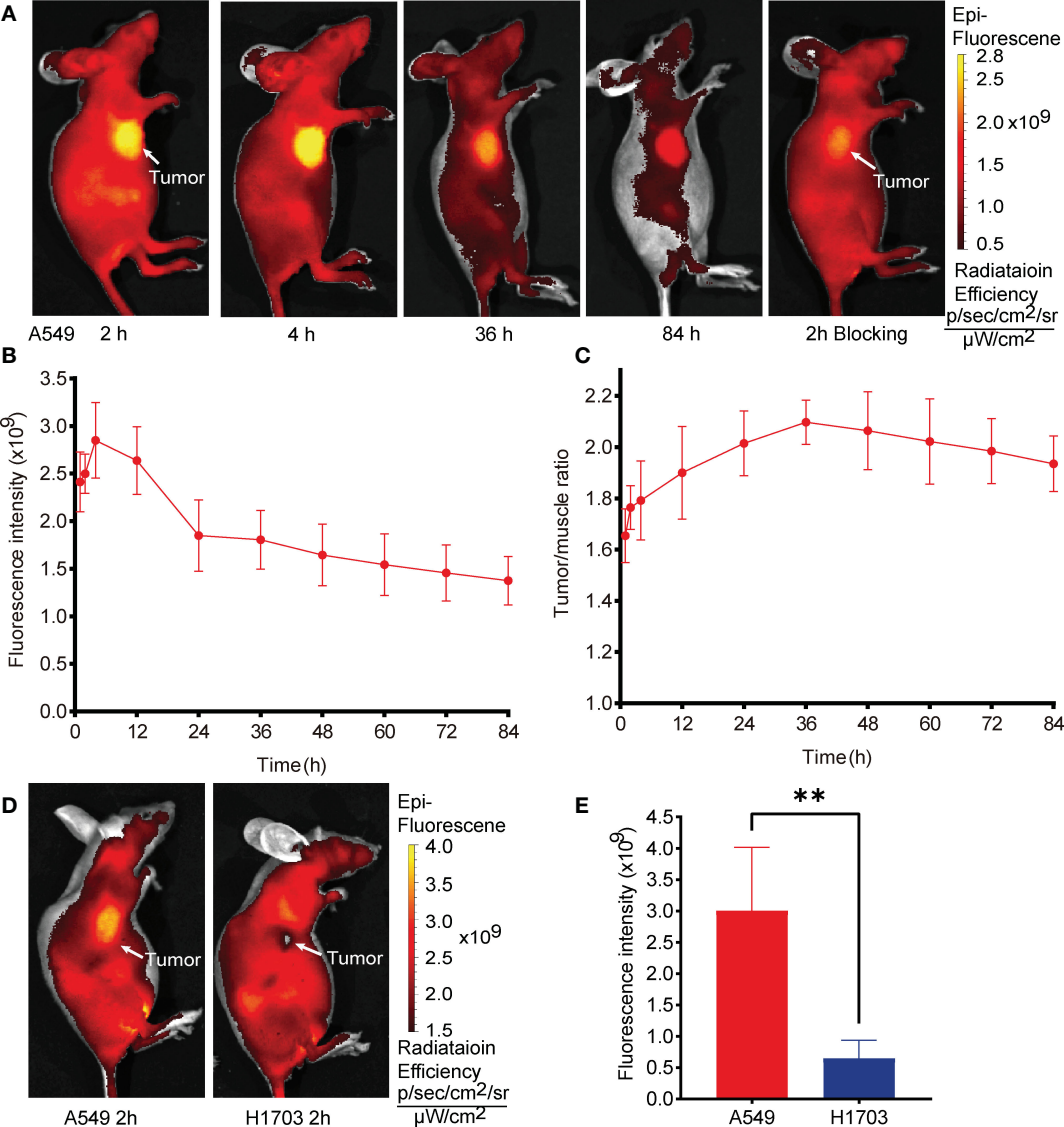
*In vivo* fluorescence images of G2-SFLAP3-Cy5.5 in subcutaneously A549 and H1703 xenograft models. **(A)** The near-infrade fluorescence images of A549 bearing mice models at different time points after injection of G2-SFLAP3-Cy5.5. **(B, C)** The quantitative average fluorescence intensity and tumor/muscle signal ratio at different time points (n=4). **(D)** Comparison of G2-SFLAP3-Cy5.5 uptake between A549 and H1703 tumors. **(E)** The corresponding average fluorescence intensity in A549 and H1703 tumors (n=4). Data were presented as mean± SD, **p < 0.01.

The *in vivo* efficacy and metabolic characteristics of G2-SFLAP3-Cy5.5 were analyzed in subcutaneous xenograft models. **Figure 2A** showed the fluorescence images of A549 xenograft models after intravenous injection of 1nmol G2-SFLAP3-Cy5.5. The average fluorescence intensity of tumors gradually increased at early time points (1h, 2h), peaked at 4h p.i., and gradually decreased subsequently (**Figure 2B**). It indicated that G2-SFLAP3-Cy5.5 could rapidly accumulate in the tumor as early as 1h p.i, and the tumor fluorescence signal remained clearly visible at 84h p.i. The signal/background ratios (SBR) between tumors and muscle were calculated each time. Since the background signal dropped faster, the SBR continued to rise from 0 to 36 h and reached the highest point, measured as 2.10 ± 0.087 (mean ± SD); then, the SBR gradually decreased and maintained 1.93 ± 0.109 at 84 h p.i (**Figure 2C**). The tumor-targeting specificity of G2-SFLAP3-Cy5.5 was confirmed through comparison with H1703 xenograft models and blocking experiments. The uptake of G2-SFLAP3-Cy5.5 can be blocked by excess G2-SFLAP3 in A549 xenograft models (**Figure 2A**). H1703 tumors, with low expression of integrin αvβ6, showed significantly lower uptake of G2-SFLAP3-Cy5.5 than A549 (p<0.01) (**Figures 2D, E**).

**Figure 3:**
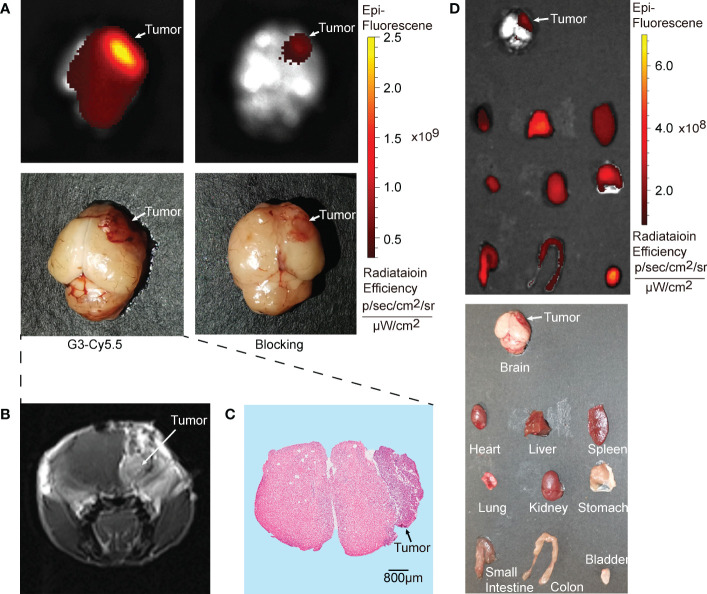
*Ex vivo* fluorescence images of G2-SFLAP3-Cy5.5 in A549 brain metastasis models. **(A)** The NIR images of G2-SFLAP3-Cy5.5 and blocking experiment for brain metastatic A549 at 2 hours post-injection. **(B)** Enhanced brain MRI scan for brain metastasis xenograft model. **(C)** HE staining of brain slices. **(D)** The *ex vivo* images of G2-SFLAP3-Cy5.5 at 2 hours post-injection through fluorescence and visible light.

The intracranial efficacy of G2-SFLAP3-Cy5.5 was analyzed through *ex vivo* fluorescence images of brain metastasis xenograft models. G2-SFLAP3-Cy5.5 could specifically aggregate in intracranial A549 tumors and showed high contrast to the surrounding brain tissue (**Figure 3A**). The imaging localization of intracranial tumors was consistent with MRI (**Figure 3B**) and hematoxylin and eosin (HE) staining (**Figure 3C**). Mice in the blocking group showed decreased fluorescence signal at 2h p.i. (**Figure 3A**). These findings suggested that G2-SFLAP3-Cy5.5 can pass through the blood-brain barrier and specifically bind to brain metastatic A549 lesions.

The *ex vivo* imaging was performed at 2h p.i. to acquire the fluorescent images of organs. The bladder showed the highest fluorescence signal, followed by the liver. The kidney, heart, spleen, lung, and gastrointestinal tract also had moderate G2-SFLAP3-Cy5.5 accumulation. The brain showed a low fluorescence intensity, which provided a clean background for brain metastasis imaging (**Figure 3D**).

### 3.3 PET imaging

**Figure 4:**
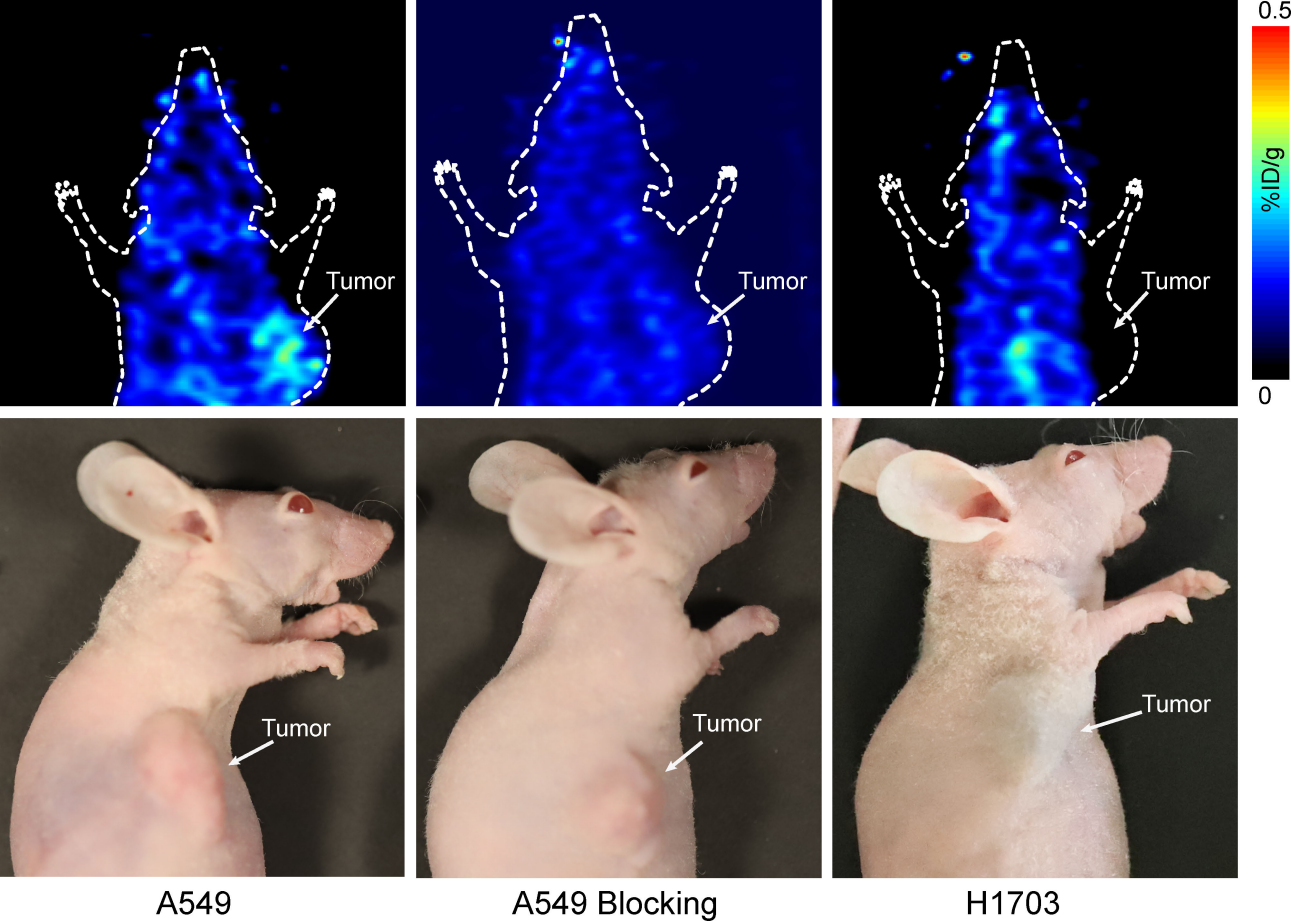
Small-animal PET image of 68Ga-DOTA-G2-SFLAP3 for mice with A549, H1703, and G2-SFLAP3-blocked A549.

As shown in **Figure 4**, the A549 tumors were visualized with good tumor/background ratio at 20 min p.i. of 68Ga-DOTA-G2-SFLAP3. On the contrary, H1703 and A549-blocking tumors did not show obvious uptake of 68Ga-DOTA-G2-SFLAP3.

## 4 Discussion

Integrin αvβ6 has been proven to be a good candidate for tumor-targeted imaging and treatments because it was highly expressed in various malignancies and absent or weak in normal tissue (22–24). Several preclinical radiotracers targeting integrin αvβ6 have been developed for PET imaging. A20FMDV2 was the first generation αvβ6-targeting agent; however, rapid tumor wash-out and poor *in vivo* stability limited its further application. In 2017, sunflower trypsin inhibitor (SFTI)-1 based peptide SFITGv6 was developed. The PET/CT scan of NSCLC and HNSCC suggested SFTIGv6 accumulated specifically in tumors rather than inflammatory lesions. In 2018, Roesch developed the SFTI1-based peptide SFLAP3, which contained latency-associated peptides 3 (16). SFLAP3 had a better affinity to integrin αvβ6 than SFITGv6. The PET/CT scan of a patient with HNSCC showed specific SFLAP3 accumulation in the primary tumor (SUVmax, 5.1) and lymph node metastases (SUVmax, 4.1) (16). The novelty of the current study was that we synthesized a G2-SFLAP3 peptide based on the structure SFLAP3 and labeled G2-SFLAP3 with Cy5.5 and 68Ga; and validated the *in vivo* tumor-targeting efficacy of G2-SFLAP3. Our results showed the uptake of G2-SFLAP3 by tumor cells was consistent with the expression of integrin αvβ6. The fluorescent and radioactive uptake of G2-SFLAP3 was specifically detected in integrin αvβ6-positive tumors. Furthermore, G2-SFLAP3 could pass through the blood-brain barrier and detect brain metastases.

The upregulated integrin αvβ6 could promote tumor metastasis (7, 21, 25). Previous studies also found that integrin αvβ6-targeted PET could accurately detect primary and metastatic tumors (26, 27). In the current study, G2-SFLAP3-Cy5.5 can detect brain metastatic NSCLC with high signal-background ratio. It was not surprising because the previous immunohistochemistry test has shown integrin αvβ6 was absent in normal brain (28) and highly expressed in 53.9% (103/191) of brain metastatic lung cancer (12). At present, the mainstem PET agent is [18F]-FDG; however, the high glucose metabolism in normal brain tissue limits its diagnostic accuracy for brain tumors (29). Moreover, a previous study has demonstrated integrin αvβ6 was absent from gliomas (28). Accordingly, 68Ga-DOTA-G2-SFLAP3 is a promising radiotracer for early detection of brain metastases and differential diagnosis with primary brain tumors.

Radical resection of tumors without causing apparent iatrogenic damage is crucial for improving patients’ prognosis and life quality. By conjugating NIR dyes with tumor-targeting agents, NIR fluorescence optical imaging can detect tumors and guide surgeries (30, 31). In the current research, G2-SFLAP3-Cy5.5 showed high and specific accumulation in integrin αvβ6-positive tumor lesions; and had potential value for intraoperative surgical guidance in patients.

In the current study, G2-SFLAP3-Cy5.5 showed a long fluorescent imaging time, as long as 84 hours. It reflected the *in vivo* stability of G2-SFLAP3. In general, the imaging window of αvβ6-targeting polypeptide radiotracers was relatively short, usually less than 6 hours (16, 17, 22, 32). We speculated that connecting G2-SFLAP3 with lipophilic Cy5.5 prolonged its metabolic time. For example, the *ex vivo* image showed that G2-SFLAP3-Cy5.5 was mainly excreted through the urinary system, while the liver also had a slightly high accumulation of G2-SFLAP3-Cy5.5. In addition, G2-SFLAP3-Cy5.5 can pass through the blood-brain barrier, with a molecular weight of 1841 g/mol. And it was well known that lipophilic molecules are more likely to penetrate the blood-brain barrier (33). Therefore, all this evidence suggested that G2-SFLAP3-Cy5.5 had considerable lipophilicity. On the other hand, our experiment showed that 68Ga-DOTA-G2-SFLAP3 had a good water solubility, which was consistent with previous studies (16, 17). 68Ga, with a relatively short half-life, can still reflect the metabolic characteristics of G2-SFLAP3.

There is increasing evidence for integrin αvβ6 as a prognostic biomarker (7, 27, 34). Elayadi performed a retrospective study on 311 lung cancer (293 NSCLC, and 18 small cell lung cancer) and found that the up-regulation of integrin αvβ6 in lung cancer was associated with poor prognosis (11). Thus, αvβ6-targeted G2-SFLAP3 had a potential role for prognostic evaluation. Furthermore, since the specific expression of integrin αvβ6 in malignancies, integrin αvβ6 may be a potential therapeutic target. For example, SFALP3 could be applied for peptide receptor radionuclide therapy (PRRT) through labeling with a therapeutic isotope. A dural or multi-functional molecular imaging agent composed of both a radioisotope and a NIR dye could also be synthesized. Further work needs to be done to explore the therapeutic effect of G2-SFLAP3 on NSCLC.

However, the current study still had some limitations. First, we used human NSCLC xenograft mice models, which cannot fully mimic the clinical situation. Further studies using patient-derived xenograft (PDX) models may be needed to demonstrate the potential of G2-SFLAP3 for clinical use. In the current study, we mainly focused on the imaging performance of G2-SFLAP3; however, its biosecurity such as stimuli responsiveness, serum stability, and toxicological effect should be further studied. Furthermore, there was lacked comparison between different imaging agents, although several integrin αvβ6-targeting probes had been developed previously. Further studies need to be done to select the integrin αvβ6-targeting agent with better binding affinity and specificity. Moreover, the number of brain metastatic mouse models was limited, and these results needed further quantitative verification.

## 5 Conclusions

We have successfully synthesized the Cy5.5 labeled and 68Ga labeled integrin αvβ6-targeting tracer G2-SFLAP3, and tested the imaging efficacy in both NIR fluorescence and PET modalities. G2-SFLAP3-Cy5.5 could selectively image integrin αvβ6-positive NSCLC xenograft mice models, and detect brain metastasis through the blood-brain barrier. 68Ga-DOTA-G2-SFLAP3 can also specifically detect integrin αvβ6-positive tumors. G2-SFLAP3 could be applied for noninvasive imaging and intraoperative guidance of NSCLC and brain metastases.

## Data availability statement

The datasets presented in this study can be found in online repositories. The names of the repository/repositories and accession number(s) can be found in the article/**Supplementary Material**.

## Ethics statement

The animal study was reviewed and approved by the ethical committee of Beijing Tiantan Hospital.

## Author contributions

DF and CZ contributed equally to this work. All authors contributed to the study conception and design. Material preparation, data collection and analysis were performed by DF, CZ and BL. The first draft of the manuscript was written by CZ and all authors commented on previous versions of the manuscript. All authors contributed to the article and approved the submitted version.
