# Supplementary material for: In vivo evaluation of integrin αvβ6-targeting peptide in NSCLC and brain metastasis
Source: Front Oncol. 2023 Mar 10;13:1070967. doi: 10.3389/fonc.2023.1070967 (PMC10036820; doi:10.3389/fonc.2023.1070967)
Supplement: Supplementary file 1 [file Image_1.pdf]

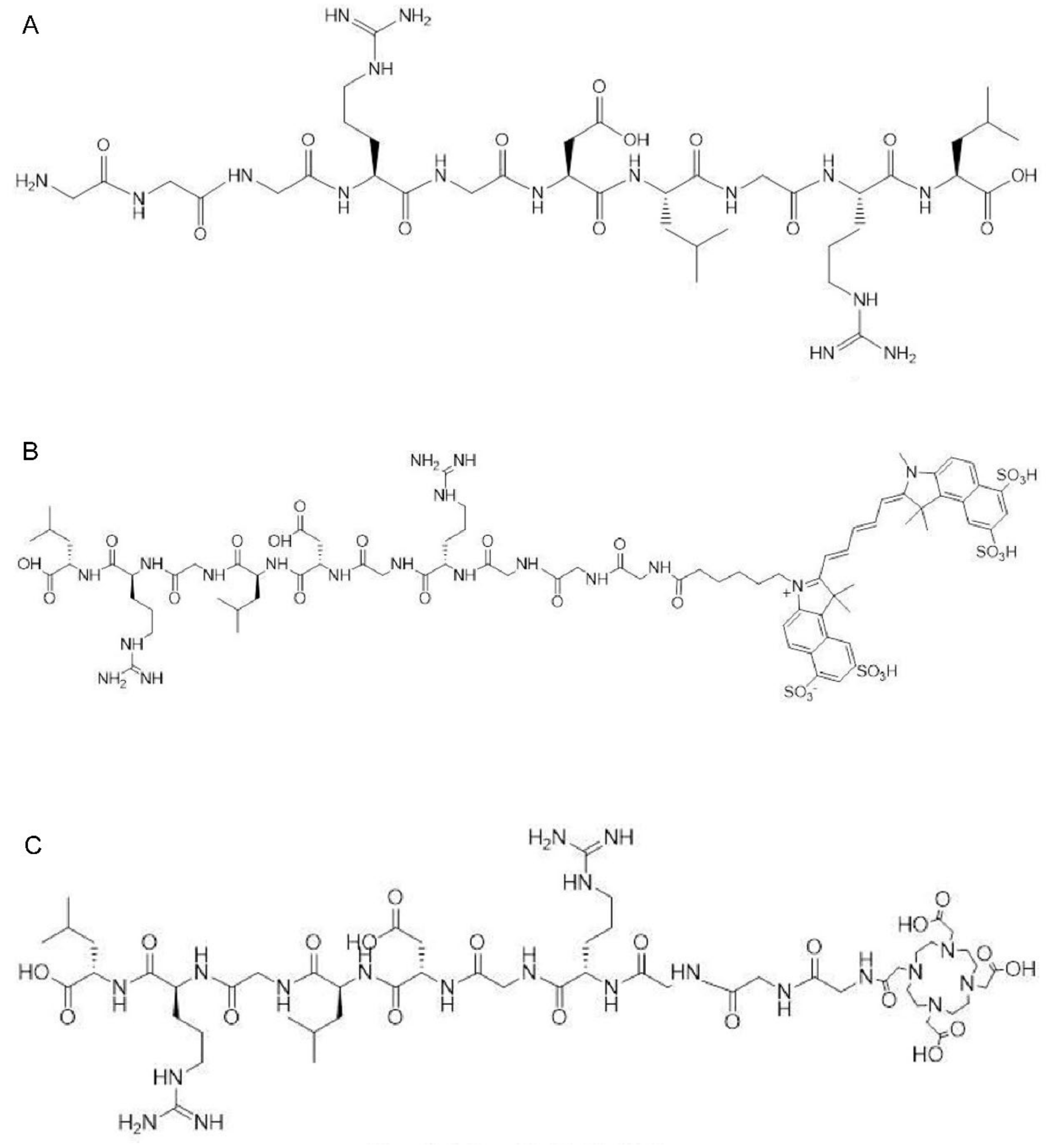

Figure S1. The structure of **(A)** peptide G2-SFLAP3, **(B)** G2-SFLAP3-Cy5.5, and **(C)** DOTA-G2-SFLAP3.
